# Associations of BMI, waist circumference, body fat, and skeletal muscle with type 2 diabetes in adults

**DOI:** 10.1007/s00592-019-01328-3

**Published:** 2019-03-29

**Authors:** T. S. Han, Y. Y. Al-Gindan, L. Govan, C. R. Hankey, M. E. J. Lean

**Affiliations:** 1 Institute of Cardiovascular Research, Royal Holloway, University of London, Egham, UK; 2 Department of Diabetes and Endocrinology, Ashford and St Peter’s NHS Foundation Trust, Chertsey, UK; 3 Department of Nutrition, School of Medicine, University of Glasgow, Glasgow, UK; 4 Department of Clinical Nutrition, Imam Abdulrahman bin Faisal University, Dammam, Saudi Arabia; 5 Health Economics and Health Technology Assessment, Institute of Health and Wellbeing, University of Glasgow, Glasgow, UK

**Keywords:** Anthropometry, Obesity, Health surveys, Adiposity

## Abstract

**Aims:**

Type 2 diabetes (T2D) is known to be associated with high BMI and waist circumference (WC). These measures do not discriminate well between skeletal muscle (SM) and body fat (BF), which may have opposite influences.

**Methods:**

We conducted a secondary analysis of population-based data from 58,128 aged 18–85 yrs from Scottish Health Surveys (2003, 2008–2011) and Health Surveys for England (2003–2006, 2008–2013), excluding pregnant women and insulin-treated diabetes. Logistic regression was used to assess associations of known T2D, and of screened HbA1c > 48 mmol/mol (> 6.5%), with sex-specific quintiles of BMI, WC, and BF% and SM% estimated by validated anthropometric equations, adjusted for age, sex, smoking, ethnicity, country, and survey year.

**Results:**

As expected, ORs for having known T2D rose with quintiles of BMI (1, 1.5, 2.3, 3.1, and 6.5) and WC (1, 1.8, 2.5, 3.5, and 8.7). Compared to the lowest BF% quintile, OR for having T2D in highest BF% quintile was 11.1 (95% CI = 8.4–14.6). Compared to the highest SM% quintile, OR for having T2D in lowest SM% quintile was 2.0 (1.7–2.4). Of 72 adults with T2D/HbA1c > 6.5% in the lowest quintile of BF%, 27 (37.5%) were in quintile 1 of SM%. Similar patterns of OR were observed for having HbA1c > 6.5% in those without known T2D.

**Conclusions:**

Estimated BF% associates strongly with T2D. Low SM% also has a significant association, suggesting a neglected aspect of aetiology within T2D. These two simple measures with biological relevance, available from data collected in most health surveys, may be more useful than the purely statistical terms BMI.

## Introduction

Type 2 diabetes (T2D) is known to be associated with a number of anthropometric indices of adiposity including body mass index (BMI), waist circumference (WC), and waist-to-hip ratio [1]. It is generally accepted that increased adiposity is causal in people who are (epi-)genetically predisposed to metabolic syndrome, but T2D can occur in people of normal BMI. While extreme BMI, e.g., > 35 kg/m^2^, always indicates excess body fat, but does not relate to specific body compartments; therefore, this index should not be applied to individuals, while its use in general populations can be misleading [2–5]. WC is an alternative which is more specifically associated with total body and distribution of body fat than BMI [6]. In most studies [7, 8], but not all [9, 10], WC has been shown to have somewhat stronger association than BMI with the development of T2D and other cardiometabolic disorders. In contrast, hips circumference (HC) has an inverse relationship with metabolic diseases [11]. A large HC may be protective, because it reflects a greater insulin-sensitive gluteo-femoral muscle mass, while smaller HC may indicate gluteo-femoral muscle atrophy [12].

In principle, increased body fat is likely to promote T2D by impairing insulin sensitivity, and possibly insulin secretion, particularly when there is ectopic fat in the liver, muscle, and pancreas [13]. However, the main organ for glucose disposal, and fat oxidation, is skeletal muscle, so decreased muscle mass might also be expected to promote T2D, and conditions with muscle loss or atrophy do exhibit impaired glucose tolerance [14].

Accurate measurement of body composition requires complex methods that are not practically applicable in large surveys. Some indirect field methods such as bioelectrical impedance have been used in large studies, but they gain little or no advantage over anthropometry, when compared against reference methods such as magnetic resonance imaging (MRI) [15]. Most epidemiological studies of metabolic disorders use BMI, for gauging body composition, but BMI does not discriminate well between skeletal muscle (SM) and body fat (BF), which may have opposite influences on T2D development.

The present study explores the associations of T2D and glycated haemoglobin (HbA1c) with BF and SM calculated from equations validated against MRI, in a large database of national health surveys.

## Subjects/methods

### Study design, patients, and setting

**Fig. 1:**
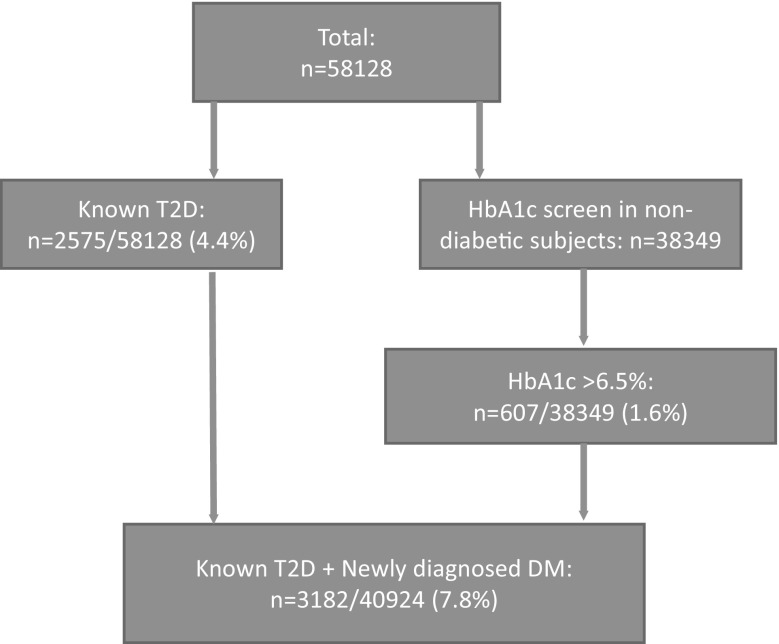
Flowchart showing the numbers of subjects available for analysis in the present study

This analysis utilized cross-sectional data from the Scottish Health Survey (SHS) collected in 2003 and 2008–2011 (*n* = 92,216) and Health Survey for England (HSE) collected in 2003–2006 and 2008–2013 (*n* = 140,627). The surveys followed identical methods. Subjects younger than 18 years or over 85 years were excluded, because they were outside the age range of populations used to derive the equations for estimating BF and SM. Pregnant women and patients with insulin-treated diabetes were also excluded, leaving 58,128 (26,292 men and 31,836 women) with complete data for anthropometric, BF, SM, and T2D, and 38,349 with HbA1c screened in individuals without a previous diagnosis of T2D (Fig. 1).

### Anthropometry

Participants were visited at home by trained nurses who recorded demographic information including age, sex, ethnicity, smoking status, medical history, and treatment by standard health and lifestyle questionnaires. Smoking status was reported in categories (never smoked, used to smoke occasionally, used to smoke regularly, and current smoker). The trained nurses also measured weight, height, and waist and hip circumferences by calibrated instruments. Participants were asked to wear light clothing and stand straight in a relaxed position, feet 25–30 cm apart. WC was measured midway between the iliac crest and lowest rib, and HC at the largest circumference around the buttocks.

### Body fat and skeletal muscle calculations

Percentage body fat was calculated using validated published equations for men: BF% = 0.567 × WC (cm) + 0.101 × age (years)−31.8 and for women: BF% = 0.439 × WC (cm) + 0.221 × age (years)−9.4 [2]. SM was calculated using published validated equations for men: SM (kg) = 39.5 + 0.665 × body weight (kg)−0.185 WC (cm)−0.418 × HC−0.08 × age (years), and for women: SM (kg) = 2.89 + 0.255 × weight (kg)−0.175 × HC (cm)−0.038 × age (years) + 0.118 × height (cm) [16]. SM was expressed as percent body weight for analysis.

### Outcome variables

HbA1c was measured using non-fasting blood samples. Diabetes mellitus, assumed to be T2D as insulin-treated subjects were excluded, was identified first from participants reporting that the diagnosis had been confirmed by a doctor or a nurse, or secondly as newly diagnosed on the basis of having HbA1c > 48 mmol/mol (> 6.5%) without previously diagnosed T2D.

### Statistical analysis

Group differences were assessed by independent t test for continuous variables and by chi-squared test for categorical variables. The associations of T2D or HbA1c > 6.5% (dependent variables) with BMI, WC, BF, and SM (independent variables) were assessed by logistic regression analysis to estimate odds ratios (ORs) and 95% confidence intervals (CIs). Independent variables were categorised into quintiles. Data were adjusted for age, sex, smoking status (categorised into non-smokers and current or ex-smokers), ethnicity (categorised into white Caucasians or others), survey year, and country. Analyses were conducted using SPSS (version 23.0). The null hypothesis was rejected when *P* < 0.05.

## Results

Men and women had similar mean (± SD) age (50.2 years ± 18.8 v.s. 49.3 years, ± 18.7), BMI (27.8 kg/m^2^ ± 4.5 v.s. 27.3 kg/m^2^, ± 5.6), and HC (103.9 cm ± 10.4 v.s. 103.8 cm ± 10.1). Men were taller (174.6 cm ± 7.2 v.s. 161.2 cm ± 6.8, *P* < 0.001) and heavier (84.1 kg ± 14.9 v.s. 70.8 cm ± 14.9, *P* < 0.001), and had larger WC (98.1 cm ± 12.2 v.s. 87.6 cm ± 13.2, *P* < 0.001), SM (29.8 kg ± 6.2 v.s. 19.9 kg, ± 3.0, *P* < 0.001), and SM% (35.6% of body weight ± 5.4 v.s. 28.7% of body weight, ± 4.3, *P* < 0.001), while women had higher BF than men (29.1 kg ± 11.5 v.s. 25.1 kg, ± 8.3, *P* < 0.001) and BF% (40.0% of body weight ± 7.6 v.s. 28.9% of body weight, ± 7.7, *P* < 0.001).

Table 1 shows distribution of demographic factors and prevalences of T2D and of HbA1c > 6.5%. The prevalence of T2D increased with age, and it was higher in men than in women, in current and ex-smokers than in non-smokers, in ethnic minorities than in white Caucasians, and in Scotland than in England.

**Table 1:** Distribution of T2D and HbA1c > 6.5% among demographic factors

	T2D (*n* = 2575)			HbA1c > 6.5% without known T2D (*n* = 607)		
	*n* (% of study cohort)	Prevalence (%)	Chi-square test	*n* (% of study cohort)	Prevalence (%)	Chi-square test
Age (years)						
18–29	8574 (1.8%)	0.4%	< 0.001	5097 (13.3%)	0.2%	< 0.001
30–39	10,428 (17.9%)	0.9%		6885 (18.0%)	0.3%	
40–49	11,408 (19.6%)	2.2%		7913 (20.6%)	0.9%	
50–59	10,565 (18.2%)	4.7%		7216 (18.8%)	2.0%	
60–69	9621 (16.6%)	8.7%		6509 (17.0%)	2.8%	
70–79	6067 (10.4%)	12.0%		3859 (10.1%)	3.8%	
80–85	1465 (2.5%)	9.2%		870 (2.3%)	3.7%	
Sex						
Men	26,292 (45.2%)	5.5%	< 0.001	17,425 (45.4%)	1.9%	< 0.001
Women	31,836 (58.8%)	3.6%		20,924 (54.6%)	1.3%	
Smoking status						
Non-smokers	15,959 (27.5%)	3.6%	< 0.001	10,567 (27.6%)	1.5%	0.139
Current and ex-smokers	42,124 (72.5%)	4.7%		27,759 (72.4%)	1.6%	
Ethnicity						
White Caucasians	50,106 (86.2%)	4.2%	< 0.001	33,439 (87.4%)	1.5%	0.001
Others	8022 (13.8%)	5.8%		4910 (12.8%)	2.1%	
Country						
England	48,760 (83.9%)	4.2%	< 0.001	31,442 (82.0%)	1.5%	0.043
Scotland	9368 (16.1%)	5.6%		6907 (18.0%)	1.8%	

Tables 2 and 3 show that adjusted ORs for having T2D increased with increasing sex-specific quintiles of BMI, WC, and BF%, and with decreasing SM% (decreasing muscularity). Prevalences of T2D within quintiles of BF% were 0.5, 1.4, 2.6, 5.1, and 12.5% and conversely within quintiles of SM% were 10.1, 5.3, 3.0, 1.9, and 1.8%. Compared with the lowest BMI or WC quintile, OR for having T2D in the highest BMI quintile was 6.5 (5.5–7.8) and in the highest WC quintile was 8.7 (7.0–10.7). Compared to the lowest BF% quintile, OR for having T2D in highest BF% quintile was 11.1 (95% CI = 8.4–14.6). BF% was associated with T2D more strongly than either BMI or WC throughout all ranges of BMI including among those with BMI < 25 kg/m^2^. Compared to the highest SM% quintile, OR for having T2D in lowest SM% quintile was 2.0 (1.7–2.4). Of 72 adults with T2D/HbA1c > 6.5% in the lowest quintile of BF%, 27 (37.5%) were in quintile 1 of SM%.

**Table 2:** Associations of BMI and WC by sex-specific quintiles, with T2D (*n* = 2575) or with HbA1c > 6.5% but without known T2D (*n* = 607)

	Risk for having T2DM					Risk for having HbA1c > 6.5%				
	Group differences			Logistic regression^‡^		Group differences			Logistic regression^‡^	
	*n*	Prevalence	*P* ^†^	OR (95% CI)	*P*	*n*	Prevalence	*P* ^†^	OR (95% CI)	*P*
BMI quintile 1 (kg/m^2^): M < 24.0, F < 22.6 (referent)	11,625	1.3	< 0.001	1	–	7691	0.4	< 0.001	1	–
BMI quintile 2 (kg/m^2^): M = 24.0–26,2, F = 22.6–25.1	11,626	2.2		1.45 (1.18–1.78)	< 0.001	7990	0.7		1.32 (0.86–2.03)	208
BMI quintile 3 (kg/m^2^): M = 26.2–28.2, F = 25.1–27.7	11,626	3.7		2.25 (1.86–2.72)	< 0.001	8011	1.1		1.95 (1.31–2.91)	0.001
BMI quintile 4 (kg/m^2^): M = 28.2–30.9, F = 27.7–31.4	11,514	5.3		3.11 (2.58–3.74)	< 0.001	7637	2.0		3.55 (2.44–5.16)	< 0.001
BMI quintile 5 (kg/m^2^): M ≥ 30.9, F ≥ 31.4	11,737	9.6		6.54 (5.48–7.80)	< 0.001	7020	4.0		7.77 (5.42–11.13)	< 0.001
WC quintile 1 (cm): M < 88.0, F < 76.0 (referent)	11,618	0.8	< 0.001	1	–	7770	0.3	< 0.001	1	–
WC quintile 2 (cm): M = 88.0-94.6, F = 76.6–82.7	11,577	2.0		1.83 (1.43–2.33)	< 0.001	8007	0.4		0.95 (0.55–1.62)	0.837
WC quintile 3 (cm): M = 94.6-100.5, F = 82.7–89.5	11,647	3.2		2.53 (2.01–3.18)	< 0.001	8011	1.2		2.60 (1.65–4.09)	< 0.001
WC quintile 4 (cm): M = 100.5-107.9, F = 89.5–98.5	11,637	4.9		3.53 (2.82–4.40)	< 0.001	7659	2.0		4.18 (2.70–6.46)	< 0.001
WC quintile 5 (cm): M ≥ 107.9, F ≥ 98.5	11,649	11.1		8.68 (7.01–10.74)	< 0.001	6902	4.5		9.71 (6.37–14.80)	< 0.001

^†^Chi-square test for group difference

^‡^Adjusted for age, sex, smoking, ethnicity, survey year, and country

**Table 3:** Associations of BF% and SM%, by sex-specific quintile, with T2D or with HbA1c > 6.5% but without known T2D

	Risk for having T2DM					Risk for having HbA1c > 6.5%				
	Group differences			Logistic regression^‡^		Group differences			Logistic regression^‡^	
	*n*	Prevalence	*P* ^†^	OR (95% CI)	*P*	*n*	Prevalence	*P* ^†^	OR (95% CI)	*P*
BF% quintile 1 (% weight): M < 22.6, F < 33.1 (referent)	11,625	0.5	< 0.001	1	–	7533	0.2	< 0.001	1	–
BF% quintile 2 (% weight): M = 22.6–27.0, F = 33.1–37.8	11,626	1.4		1.81 (1.34–2.46)	< 0.001	7971	0.4		1.61 (0.83–3.12)	0.161
BF% quintile 2 (% weight): M = 27.0-30.8, F = 37.8–42.0	11,627	2.6		2.81 (2.11–3.74)	< 0.001	8069	0.9		3.42 (1.87–6.26)	< 0.001
BF% quintile 4 (% weight): M = 30.8–35.2, F = 42.0-46.8	11,625	5.1		4.70 (3.55–6.22)	< 0.001	7845	1.9		6.82 (3.79–12.28)	< 0.001
BF% quintile 5 (% weight): M ≥ 35.2, F ≥ 46.8	11,625	12.5		11.05 (8.38–14.57)	< 0.001	6931	5.0		17.34 (9.70–31.00)	< 0.001
SMM% quintile 5 (% weight): M ≥ 38.3, F ≥ 31.4 (referent)	11,626	1.8	< 0.001	1	--	7171	0.5	< 0.001	1	–
SMM% quintile 4 (% weight): M = 36.1–38.3, F = 29.1–31.4	11,627	1.9		0.94 (0.77–1.15)	0.554	7749	0.6		1.12 (0.73–1.71)	0.607
SMM% quintile 3 (% weight): M = 34.1–36.1, F = 27.3–29.1	11,657	3.0		1.10 (0.92–1.33)	0.277	7893	1.2		1.69 (1.15–2.50)	0.008
SMM% quintile 2 (% weight): M = 31.7–34.1, F = 25.2–27.3	11,593	5.3		1.46 (1.22–1.74)	< 0.001	7975	1.9		2.16 (1.48–3.16)	< 0.001
SMM% quintile 1 (% weight): M < 31.7, F < 25.2	11,625	10.1		2.00 (1.67–2.39)	< 0.001	7561	3.8		3.34 (2.27–4.90)	< 0.001
SM% quintile 5 and BF% quintile 1 (referent)	7315	0.4	< 0.001	1	–	4681	0.1	< 0.001	1	–
Intermediate group	44,311	3.8		3.90 (2.66–5.72)	< 0.001	29,856	1.3		7.81 (2.89–21.15)	< 0.001
SM% quintile 1 and BF% quintile 5	6502	13.5		9.74 (6.56–14.44)	< 0.001	3812	5.3		22.40 (8.12–61.76)	< 0.001

^†^Chi-square test for group difference

^‡^Adjusted for age, sex, smoking, ethnicity, survey year, and country

Very similar BF% and SM% quintile patterns were observed for prevalences and OR of having HbA1c > 6.5% without known T2D (Tables 2, 3).

**Fig. 2:**
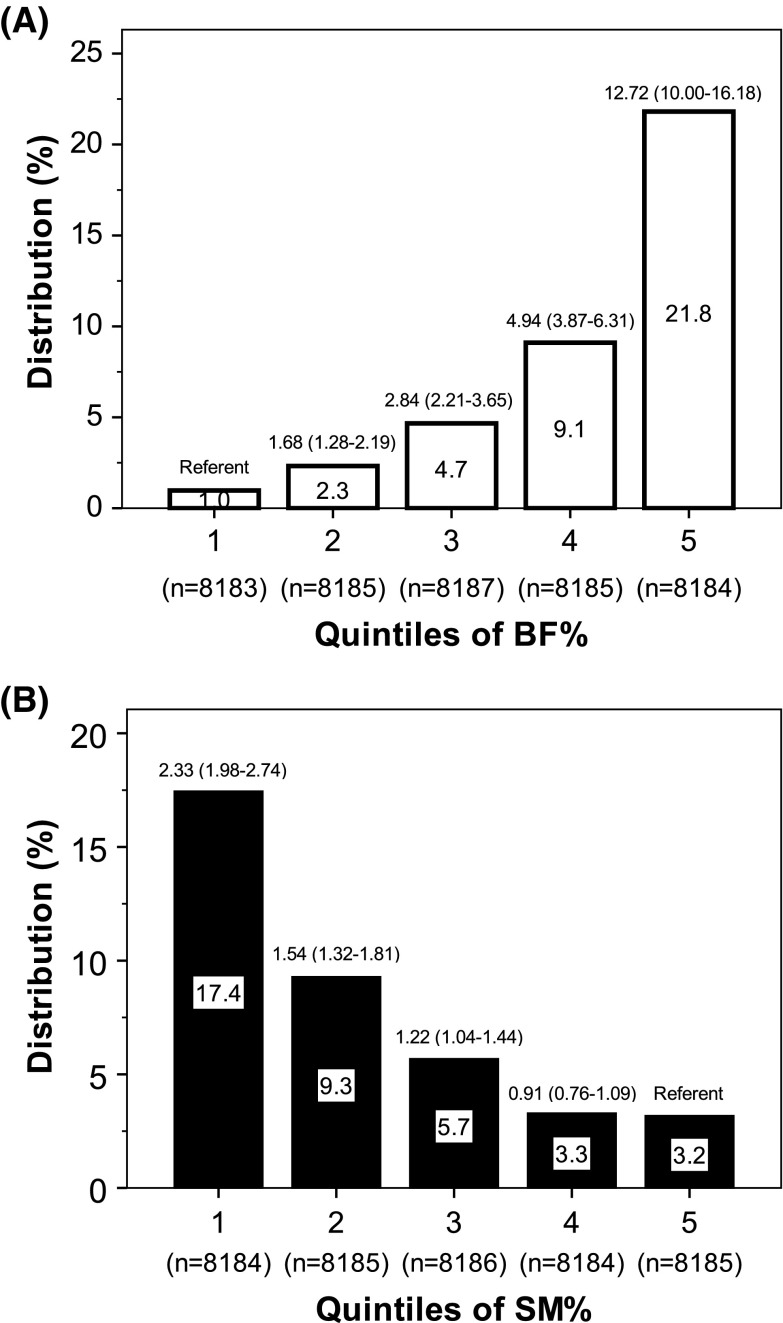
Distribution and ORs (adjusted for age, sex, smoking, ethnicity, survey year, and country) for having T2D and/or HbA1c > 6.5% in different sex-specific quintiles of BF% (**a**) or SM% (**b**) in 40,924 subjects

Further analysis was conducted by combining the individuals with known T2D and those with HbA1c > 6.5% without known diabetes, as the dependent variable. The combined prevalence of T2D again rose with higher quintiles of BF% (Fig. 2a) and with lower quintiles of SM% (Fig. 2b). Compared to individuals in the lowest sex-specific quintile of BF%, those in the highest quintile were 12.7-fold (10–16.2) more likely to have either diagnosed T2D or HbA1c > 6.5%. Compared to individuals in the highest quintile of SM%, those in the lowest quintile were 2.3-fold (2.0–2.7) more likely to have T2D or HbA1c > 6.5%. Among the 3182 of individuals with any T2D (known or new) in the lowest quintile of BF% (72), 3 (4.2%) were in the lowest quintile of SM%.

## Discussion

The present study applied published, validated, equations based on data routinely collected in most large national health surveys to estimate BF% and SM%. We confirmed the known associations of BMI and WC with HbA1c and T2D. We observed a stronger relationship between BF% and T2D, and also a significant inverse relationship between SM% and T2D. While causal relationships cannot be proved in cross-sectional studies, the enormously powerful, well-established relationship of adiposity with T2D is already accepted as its main causal factor. Weight gain which exceeds the variable ‘personal fat threshold’ reveals a predisposition, most likely epigenetic, for metabolic syndrome and T2D with ectopic (liver and pancreas) fat accumulation [13]. The inverse relationship with SM% may provide further insights into the aetiology of T2D, supporting the notion that muscle is generally protective against metabolic syndrome and T2D. This would potentially explain the protective association of high hip circumference (from gluteal muscle) [11, 12].

There was also evidence of significant associations of T2D and HbA1C > 6.5% with both low SM% and high BF%, but this combination does not always co-exist within individuals. This suggests that these two measures detect different individuals with T2D, which could point towards T2D being promoted by different body-composition phenotypes, potentially demanding different approaches to treatment and prevention.

High BF%, particularly intra-abdominal adipose tissue, may have a key intermediary role in the development of insulin resistance and subsequent T2D. It is now recognised that intra-abdominal fat is an active endocrine organ producing a number of adipocytokines such as adiponectin, leptin, resistin, and interleukins, which play a crucial role in appetite and energy regulation [14]. An imbalanced production of these factors by an expanded intra-abdominal fat mass probably contributes to weight-related metabolic disorders [17].

We have previously shown that the prevalence of metabolic syndrome was increased in those with either low BMI and high WC, or high BMI and low WC, compared to individuals who had low BMI and low WC [18]. However, a high BMI may be misinterpreted as overweight or even obesity among people in training of power sports such as American football players or heavyweight boxers, where a high BMI reflects a high SM mass [19]. This is the reason for the use of the diametrically opposed measurements of FM% and SM% herein to improve discrimination between fat and muscle components.

The significant association of HbA1c and T2D with low SM% demonstrated here has not been well documented in the literature. In a cross-sectional study of Koreans, SM was 2–4-fold lower in individuals with T2D than those without [20] and an NHANES study found inverse associations of insulin resistance index (HOMA-IR) and pre-diabetes (based on HbA1c) with a ‘skeletal muscle index’ derived from bioelectrical impedance, adjusted for age, sex, ethnicity, and adiposity [21]. It is not possible to define the mechanisms, or even direction of causality, between SM% and HbA1c or T2D, likely involving multiple factors. SM commonly falls during the clinical presentation of T2D as a direct result of diminished insulin activity [22], but it is also the main oxidative organ and site of glucose disposal. Low SM% by mass is likely to be a very crude correlate of the whole-body metabolic functions of different muscle components. T2D has been associated with a relative paucity of type 1 (oxidative) muscle fibres, and excess of type 2 muscle fibres [23]. Muscle mass may be low or reduced for genetic reasons, and by inflammatory diseases as well as malnutrition. An association between impaired glucose tolerance and T2D with low SM has been shown previously in conditions with primary muscle loss or atrophy, such as immobility [24], muscular dystrophies, myopathies [25], cancer [26], or stroke [27], while low SM has also been shown to associate with hypertension [28].

There are certain limitations to the present study, including the cross-sectional design, but that should not diminish the validity of the new analysis for BF% and SM%, particularly as the analyses for BMI and WC are in line with published data. The previous studies have shown slightly variable associations, e.g., stronger associations with WC and incidence of T2D in long term follow-up studies [29], than in cross-sectional studies where WHR tends to be more powerful. The present study adds support to the view that small hip circumference (thus higher waist:hip ratio) is predictive, because it may reflect reduced gluteal muscle mass. The equations for calculating BF% and SM% were derived in studies of predominantly Caucasian adults, but with few elderly individuals [2, 16]. The HSE and SHS databases used in the present study also comprise mostly Caucasians (86.2%), so the anthropometric equations are likely to be appropriate for their body compositions. Estimates of BF% or SM% in older people, or with different ethnic backgrounds, may be less accurate and different equations may be needed. Equations used to estimate SM include HC as one of the variables may be less accurate in older adults and in females due to their higher proportion of adipose tissue for a given HC. Our study, therefore, excluded subjects over 85 years old. However, the inclusion of this oldest age group did not substantially change our findings. The survey base was large, and surveys are typical representative national surveys, but study power was reduced, because not all the measures were made for every subject in every year.

The use of existing survey data is economical, but has drawbacks, because the number of collected variables is fixed and may limit analyses. For example, we did not have information on the duration of diabetes, but we did attempt to reduce associated errors by excluding insulin-treated patients (likely to have longer duration of diabetes). Measurement errors are always possible. Accuracy is important at individual level to avoid misclassification of subjects in categories of interest such as BMI, but the sizes of errors in measuring height, weight, or waist circumference are not great enough to cause frequent misclassification, which is more severely affected in self-reported than in measured data [30]. When analysing large national surveys to establish associations, as in the present study, minor measurement errors are unlikely to be problematic, since the large study number allows confidence in the findings. The lack of information on oral antihyperglycaemic agents is another limitation, but the patterns of associations between body composition with T2D and body composition with HbA1c are similar, suggesting a valid observation. Another common drawback with surveys is bias in participation, which may explain the relatively low prevalence of T2D in this present study, e.g., high proportion of younger adults (with low rates of T2D) and recruiting a low proportion of those with T2D because of health problems and hospital appointments, etc. Excluding those treated with insulin for the present analysis would also have lowered the apparent rates of T2D.

In conclusion, estimated BF% associates with T2D. Low SM% is also a significant factor. These two simple measures, available from data collected in most health surveys, may be more useful than using BMI or WC for assessing T2D. These results suggest two distinct body-composition phenotypes driving type-2 diabetes, with implications for prevention and treatment strategies. Further exploration would be valuable, especially confirmation in longitudinal follow-up studies.
